# Squamous Cell Proliferation as a Reactive Mechanism to Laryngeal Cryptococcus Infection: A Case Report

**DOI:** 10.7759/cureus.12587

**Published:** 2021-01-09

**Authors:** Hugh Patrick Mallany, Nikolina Curcin, Sergio Borgia, Greg Belchetz

**Affiliations:** 1 Michigan Ear Institute, Michigan State University, Detroit, USA; 2 Department of Otolaryngology, Head and Neck Surgery, William Osler Health System, Brampton Civic Hospital, Brampton, CAN; 3 Department of Otorhinolaryngology, Head and Neck Surgery, University of Pennsylvania, Philadelphia, USA; 4 Department of Pathology and Laboratory Medicine, William Osler Health System, Brampton Civic Hospital, Brampton, CAN; 5 Department of Infectious Diseases, William Osler Health System, Brampton Civic Hospital, Brampton, CAN; 6 Department of Infectious Disease, McMaster University, Hamilton, CAN; 7 Department of Otolaryngology, Head and Neck Surgery, Faculty of Medicine, University of Toronto, Toronto, CAN

**Keywords:** clinical case report, laryngeal cryptococcus, squamous cell carcinoma, fluconazole, fungal infection, pseudoepitheliomatous hyperplasia, cryptococcus neoformans (c. neoformans)

## Abstract

*Cryptococcus neoformans* (*C. neoformans) *is a ubiquitous encapsulated, obligate anaerobe, a dimorphic fungus that can be pathogenic in humans. *C. neoformans* infections arise primarily in immunocompromised individuals, such as human immunodeficiency virus (HIV) patients, as well as those who use inhaled corticosteroids regularly. Due to the wide variety of clinical findings associated with *C. neoformans,* infection within the head and neck is occasionally misdiagnosed as malignancy due to its protean manifestation. In this report, we describe the case of a *C. neoformans *infection resulting in an initial misdiagnosis due to an overlying laryngeal squamous cell proliferation mimicking laryngeal squamous cell carcinoma (SCC). This case is intended to provide further evidence for inhaled corticosteroid use predisposing patients to fungal infections, as well as to provide insight into the possible mechanism resulting in the initial diagnosis of malignancy. A review of recent literature is also discussed.

## Introduction

Laryngeal cryptococcus infections in the setting of an initial malignancy misdiagnosis is a rare clinical finding. With around 30 cases currently reported, the incidence and, therefore, the cause of this rare infection is not well understood. In this report, we describe the case of a patient presenting with chronic hoarseness of the voice who underwent a laryngeal biopsy for suspected malignancy. Initial findings suggested laryngeal squamous cell carcinoma. Upon further investigation, it was determined to be multiple fungal species found along the aryepiglottic folds. The invasive species was later diagnosed as *Cryptococcus neoformans (C. neoformans)*. The squamous proliferation was determined to be a reactive mechanism to the fungal infection, and the patient was successfully treated with fluconazole, 400 mg once daily (OD).

## Case presentation

An 83-year-old man was referred from his primary care physician to the otolaryngology service with a four-year history of chronic hoarseness (Day 0). The patient had a history of smoking, hypertension, dyslipidemia, and previous myocardial infarction resulting in stent placement. The patient also had a history of chronic asthma and chronic obstructive pulmonary disease (COPD) due to occupational exposure which was well-controlled with Spiriva® HandiHaler (tiotropium bromide), 18 mcg once daily, and Symbicort® (budesonide and formoterol fumarate dihydrate), 200 mcg two puffs twice a day (BID). Current medications also included acetylsalicylic acid - 81 mg once daily, ticagrelor - 90 mg BID, tamsulosin - 0.4 mg BID, bisoprolol - 5 mg once daily, rosuvastatin - 20 mg once daily, and an albuterol inhaler as needed. Initial otolaryngology consultation demonstrated multiple small mucosal nodules present on both vocal folds, extending onto the false cords and aryepiglottic folds bilaterally. There was significant cobblestoning in the interarytenoid area as well. Vocal cord mobility was normal, and the hypopharynx was unremarkable. The tonsils, base of tongue, pharynx, and oral cavity were also unremarkable. There was no palpable neck disease. The patient was given a diagnosis of posterior laryngitis with contributing factors likely from vocal overuse and chronic throat clearing with possible laryngopharyngeal reflux. The patient was started on an empirical trial of pantoprazole, 40 mg BID, for three months. 

**Figure 1:**
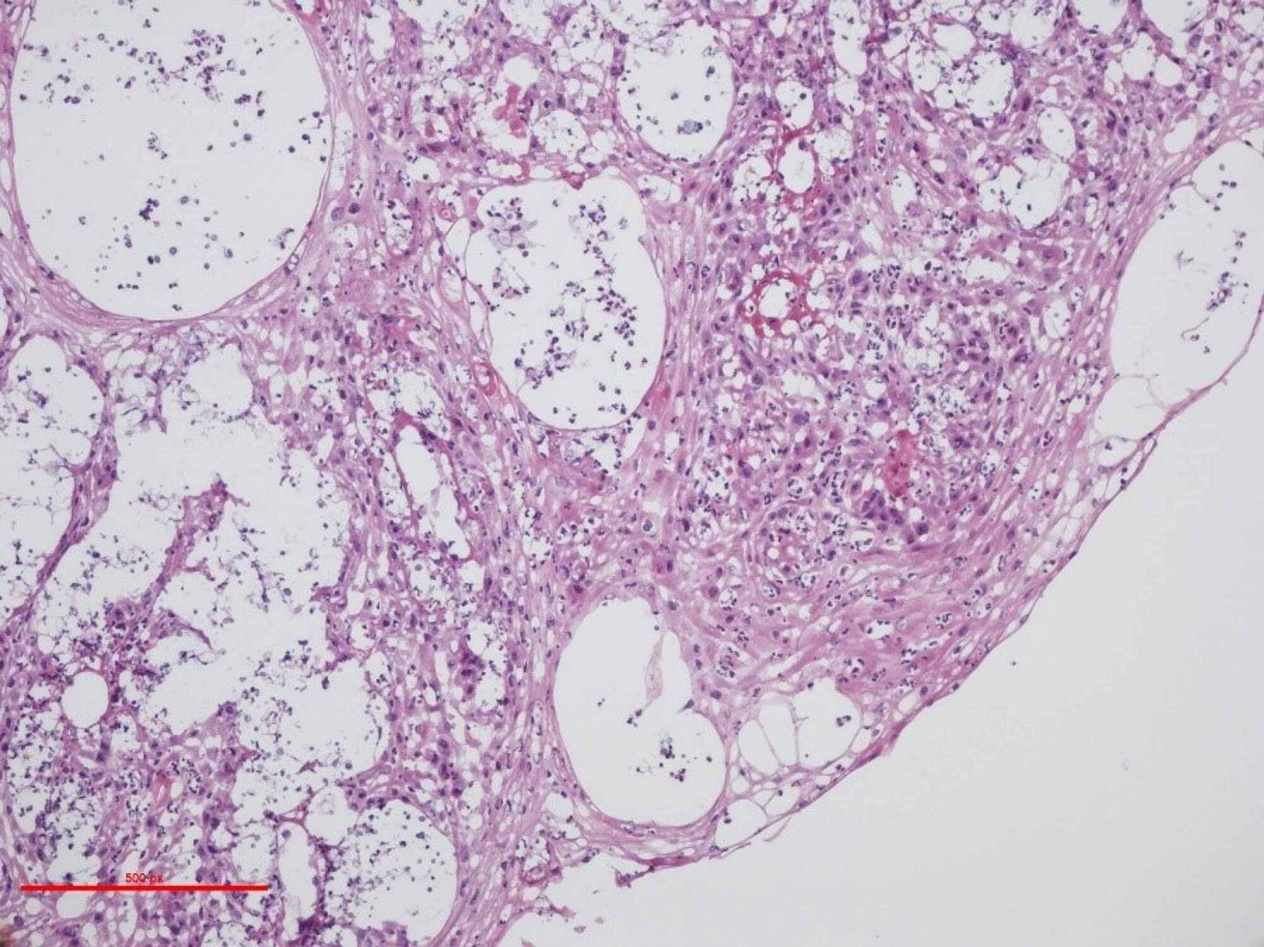
Hematoxylin and eosin (H&E) histopathology of the left aryepiglottic folds demonstrating squamous cell proliferation, acute inflammation, and scattered round-shaped microorganisms surrounded by clear halos. Magnification x100

After one month (+30 days), the patient continued to report hoarseness with minimal improvement. His pantoprazole was continued at 40 mg BID and an additional one-month follow-up was booked. On repeat nasopharyngoscopy (+60 days), the posterior glottis remained inflamed with nodules along the aryepiglottic folds bilaterally, extending into the interarytenoid area. Vocal cord mobility was normal; however, saliva pooling in the hypopharynx with laryngeal penetration was evident during this visit. Arrangements for panendoscopy with biopsy (+67 days) were made for the following week. The biopsies proceeded with two tissue samples of the left aryepiglottic folds and arytenoid, each measuring 0.3 cm and 0.4 cm. Nasopharyngoscopy showed some mucopurulent secretions coming from the sinus osteomeatal complex bilaterally, as well as the nasopharynx. No further abnormalities were noted upon examination. Sections were sent for pathological, microbiological, and frozen section analysis. Hematoxylin-eosin (H&E)-stained specimens returned with atypical squamous proliferation, acute inflammation, and scattered round-shaped microorganisms surrounded by clear halos (Figure 1). Histologic sections also demonstrated numerous thick-walled budding yeasts associated with acute inflammation overlying atypical squamous proliferation.

**Figure 2:**
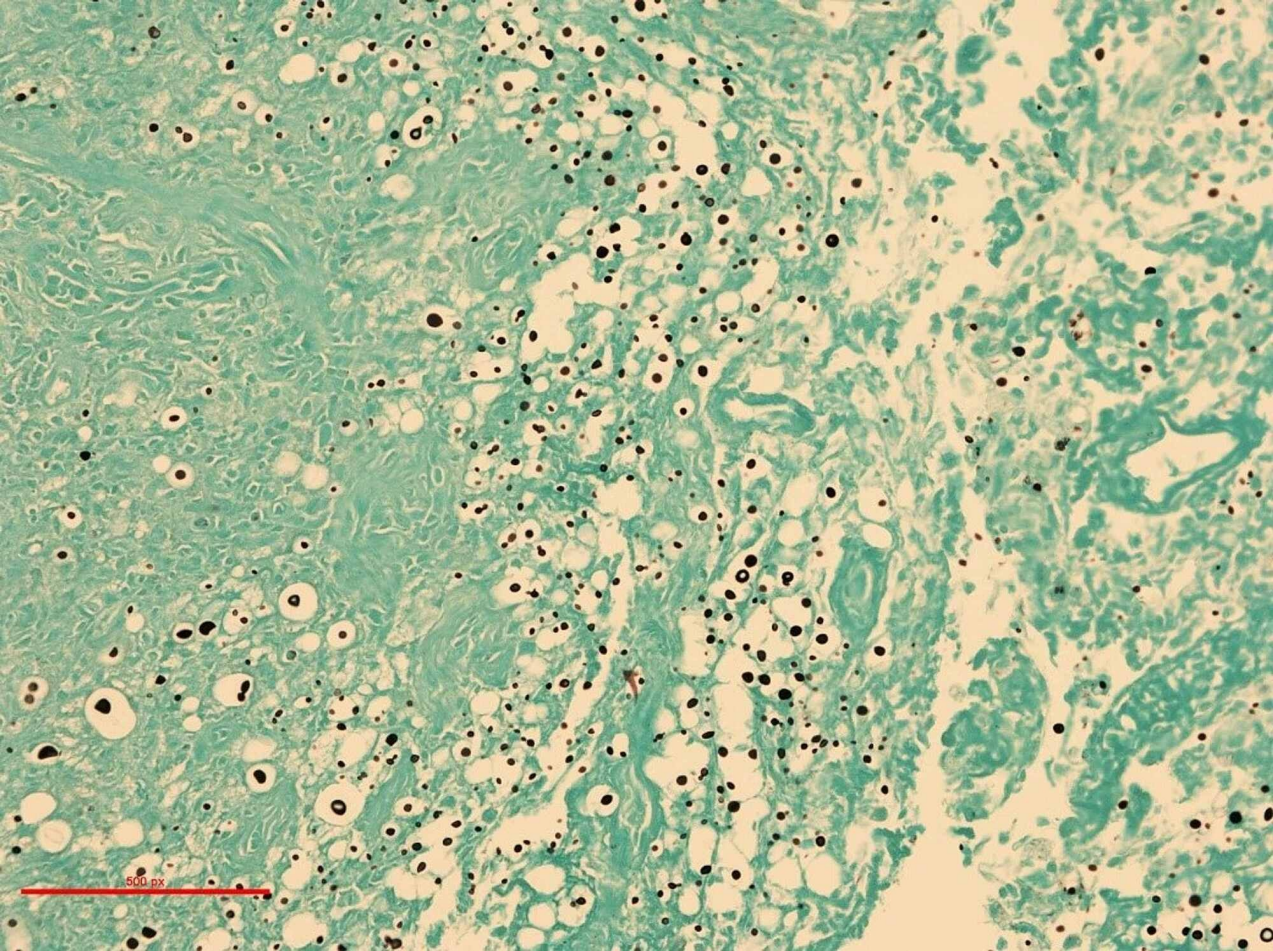
Grocott methenamine silver (GMS) histochemical stain identifying the tissue sample as positive for fungal organisms. Magnification x200

**Figure 3:**
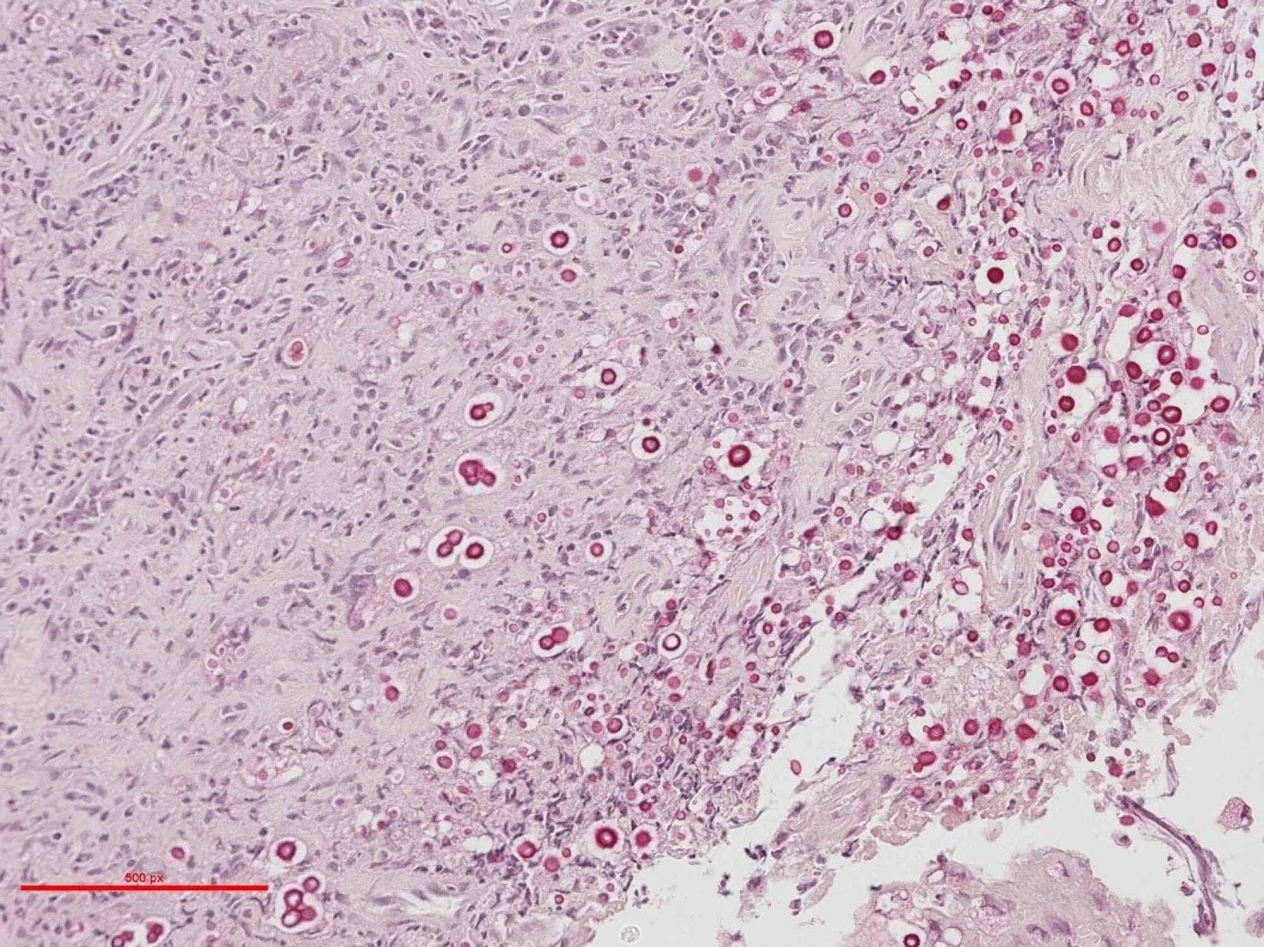
Mucicarmine periodic acid–Schiff (PAS) histochemical stain with positive mucinous capsule staining of a fungal organism. Magnification x200

The microorganisms were positive for periodic acid-Schiff (PAS) and Grocott methenamine silver stain (GMS) special stains for fungal organisms (Figure 2). Based on morphology, the major differential diagnosis consisted of *Blastomyces dermatitidis* and *Cryptococcus neoformans* infections. A special stain for mucicarmine (Figure 3) was also positive, more in keeping with the cryptococcal species. Thus, a diagnosis of atypical squamous proliferation as a reactive mechanism mediated by underlying fungal infection was postulated (0 + 75 days). The patient was referred to an infectious disease consultant for further opinion. The tissue was sent for polymerase chain reaction (PCR) analysis using universal fungal primers targeting ribosomal RNA genes. The definitive identification confirmed *C. neoformans* as the invasive organism. Additional latex agglutination was performed confirming the presence of *C. neoformans* accompanied by a titre result of 1:16.

The patient was prescribed oral fluconazole, 400 mg daily, for three months with monthly follow-up in the Infectious Disease Clinic. The patient was also referred to a respirologist where budesonide/formoterol fumarate dihydrate, two puffs BID, was discontinued. He was placed on mometasone furoate/formoterol dihydrate, two puffs BID with AeroChamber® to reduce the propensity for oropharyngeal side effects. Following three months of fluconazole therapy (+170 days), the patient’s dysphagia had resolved. On repeat nasophagyngoscopy (+177 days), the oral cavity and oropharynx were unremarkable. The nasopharynx, hypopharynx, and larynx appeared grossly normal with full resolution of aryepiglottic fold nodules. In summary, the patient had complete clinical resolution of his symptoms and was discharged cured of his infection.

## Discussion

*C. neoformans* is a saprophytic obligate aerobic encapsulated yeast typically associated with avian feces in species, such as pigeons, turkeys, and chickens. While not typically pathogenic, in the setting of the immunocompromised, *C. neoformans* possess the ability to become an opportunistic pathogen, resulting in persistent fungal infection [1]. Both primary and acquired immunodeficiencies can lead to cryptococcosis, with pulmonary infection being the most common. Extrapulmonary cryptococcal infection can occur in almost any organ; however, laryngeal *Cryptococcus* is uncommon.

Both oral and inhaled corticosteroid therapies for conditions, such as COPD, asthma, and other inflammatory diseases, predispose those individuals to infection due to suppression of cellular immune function [2]. A recent analysis of all reported laryngeal cryptococcal infections by Wong et al. described risk factors not dissimilar to our case, with this patient representing the 30th reported case of laryngeal *Cryptococcus* [3].

Of the prior 29 cases reported by Wong et al., the average age at presentation was 62 years (± 16) with no statistically significant difference between sex (52% female, 48% male). On initial consultation, all patients presented with progressive dysphonia, which is consistent with the findings of our case. Only eight (28%) of these 29 cases presented with systemic immunosuppression in the form of transplant immune suppression, therapy for inflammatory bowel disease (IBD), and rheumatoid arthritis (RA) or human immunodeficiency virus (HIV) infection. The remaining 21 cases were immunocompetent patients of which 67% were on inhaled corticosteroid therapies. While the precise mechanism of cryptococcal laryngeal infection is not well-characterized, one hypothesis postulates that the use of inhaled corticosteroid therapies results in a local immune suppression within the larynx. Accompanied with additional disruptions to the laryngeal mucosa, direct deposition and proliferation of *Cryptococcus* within the affected areas seems likely.

Interestingly, several other cases of laryngeal cryptococcosis have also reported initial suspicions of laryngeal carcinoma as the primary diagnosis prior to special fungal staining [4]. Squamous pseudoepitheliomatous hyperplasia (PEH) in the setting of fungal infection may provide a potential mechanism for the overlying squamous cell carcinoma (SCC) mimicry. PEH is a reactive epithelial proliferation that can occur secondary to different causes, including infection, neoplasm, injury, and inflammation. With the likely mechanism being cellular reactivity in the setting of a chronic inflammatory response to mediators, such as IL-1B, TNF-a, and IL6, analysis of matrix metalloproteinases, such as matrix metalloproteinase-1 (MMP-1), have also shown to be a distinguishing and measurable factor when differentiating between PEH and SCC [5]. Histologically, several features are important in distinguishing between PEH and SCC. Acanthotic epithelia accompanied by a lack of nuclear atypia and relatively few mitotic figures are generally indicative of PEH instead of SCC [6]. Furthermore, while the malignant potential of PEH is unelucidated, it is important to accurately diagnose this condition in order to avoid radical medical or surgical interventions. This case serves to highlight the importance of considering infectious causes, and in particular, fungal infections, in the differential diagnosis of laryngeal abnormalities that may masquerade as malignancy. All biopsies procured for the purpose of investigating cancer should include microbiological analyses for the presence of fungal (and other) microorganisms that are chronic, slow-proliferating, and that can cause a chronic proinflammatory mucosal response.

## Conclusions

Laryngeal cryptococcosis remains a rare but important diagnostic consideration in patients suspected of laryngeal malignancy, particularly in patients who may seem to be innocuously immunocompromised on inhaled corticosteroids for chronic pulmonary disease. This case represents only the 30th such case reported worldwide at the time written. While the exact mechanism for mycotic infection-causing pseudo-hyperplasia of the laryngeal mucosa is not fully understood, this case highlights the importance of examining laryngeal tissue for fungal infection when malignancy is suspected both to avoid the morbidity associated with misdiagnosis and given that fungal treatment is often curative and carries a favorable prognosis.
